# SENSE-L Framework: a multi-sensory pedagogical method for visually challenged learners in inclusive language and literature education

**DOI:** 10.1016/j.mex.2026.104127

**Published:** 2026-08-27

**Authors:** David Paul, Daisy Gohain, Thejas Gigy Thomas, Binu Jerline Harini S

**Affiliations:** a Department of English, School of Humanities and Social Sciences, Kristu Jayanti (Deemed to be University), Bengaluru, Karnataka, India; b School of Social Sciences and Languages, Vellore Institute of Technology, Chennai, Tamil Nadu, India; c Department of Language, Culture and Society (LCS), Faculty of Engineering and Technology, SRM Institute of Science and Technology, Kattankulathur, Tamil Nadu, India

**Keywords:** Accessibility in education, Assistive technologies, Multimodal instruction, Disability studies, Experiential learning, Tactile learning strategies, SDG, Quality education

## Abstract

This study presents the SENSE-L Framework, a multisensory systematic teaching approach to support visually challenged students in inclusive language and literature learning. The structure restructures the traditional visually-dependent teaching into a sequenced process which is based on auditory and tactile senses. It involves five sequential stages that are sensory input substitution, linguistic decomposition, sensory anchoring, guided interpretation and expressive output. The approach was confirmed by the qualitative and interview-based research with the learners and teachers, with the proportion analysis of the answers of the participants. The results show a positive understanding, recall, and learner confidence in the stages.

• Introduces an organized multisensory approach to inclusive language teaching.
• Shows staged implementation with both qualitative and quantitative validation.
• Favors the free-flowing learning in terms of auditory and tactile pedagogues.

## Graphical abstract

**Figure:**
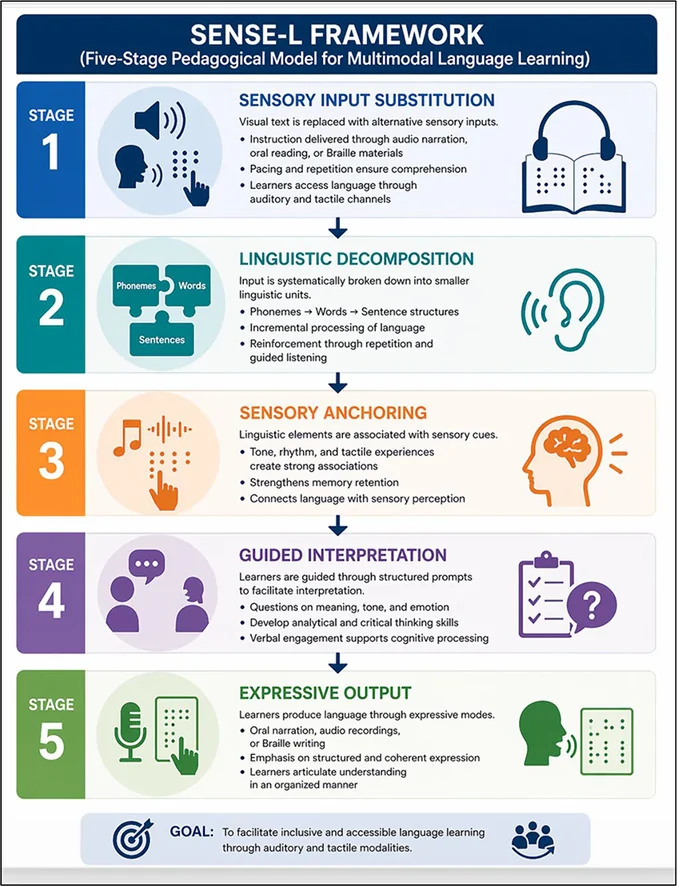

## Specifications table

**Table:** 

**Subject area**	Psychology
**More specific subject area**	Educational Psychology; Language Education; Inclusive Pedagogy
**Name of your method**	SENSE-L Framework
**Name and reference of original method**	None
**Resource availability**	None

## Background

Learning and teaching Inclusion Education has come to appreciate the need to find pedagogical models that accommodate sensorial diversity, especially to learners with visual challenges. The traditional teaching methods use a lot of visual stimulation using printed materials, written notes and education using boards that may restrict access and participation of students with visual disabilities. The studies of inclusive pedagogy repeatedly show that visually challenged learners are likely to learn better through planned auditory and tactile teaching methods, which help them not only understand but also develop their language. Innovative research by Erickson, K. A. and Hatton, D. D. While [1] indicates that ability to access alternative sensory behaviours like audio and Braille has proven to be an important step towards acquiring literacy and developing independence in learning. Similarly, Millar, S. According to [2], language decoding and cognitive organization are enhanced by tactile examination in a sequential processing. In a similar thread, recent studies have focused on the importance of Braille in literacy acquisition and inclusion by emphasizing that the combination of tactile and auditory input significantly influences the level of participation and expressive competence among students with visual impairments [3] Multisensory learning environments have also been reported to significantly stimulate the degree of In addition [4] show that the sensory substitution strategies can not only overcome the visual deficiencies, but also improve the engagement or functional learning outcomes. Collectively, these studies offer a solid empirical foundation to the idea that pedagogical frameworks that combine auditory and tactile modalities will enhance the learning process in populations with visual impairments, with systematic and organized instruction being key in overcoming challenges in learning Braille.

Multi-sensory learning, its contribution to language acquisition, and its effectiveness in improving phonological awareness and memory possessing have been greatly investigated, and it was reported that the auditory-based strategies promote phonological awareness and memory retention when reinforced with repetition and guided processing [5]. Moreover, hands-on learning, especially with the help of Braille literacy offers a necessary channel of reaching written text and forming an independent learning strategy. The methods show that language acquisition is mediated effectively by non-visual senses in case teaching is well-structured.

In literature, the inability to interpret visually necessitates learners to be more dependent on the interpretation made by hearing and the use of cognitive imaginative to form meaning. This change promotes pedagogical approaches that directly lead the students to interpretive activities so that they can interact with the tone, narration structure, and theme without a visual dependency [6]. Research on narrative experience demonstrates that sensory narration (by sound) is actively constructed, allowing readers to participate in and engage with storyworlds in more active engagement with tone, narration, and theme when visual access is diminished (Martinez M-A [7] as well as studies in multimodal and inclusive pedagogy indicate that auditory-based instruction fosters more active engagement with tone, story structure and theme. Equally, studies on reading behavior show that auditory-based/voiced reading leads to better attention to tone, prosody, and emotional effects, without which the unsupported interpretation of narrative meaning is impossible [8]. Nevertheless, the current practices are more inclined towards considering separate strategies, and they are not provided with a comprehensive methodological plan.

The SENSE-L Framework fills this gap by offering a multi-stage, structured approach to the model of pedagogical intervention by incorporating sensory substitution, linguistic processing, and expressive output. The framework enables teachers to provide an inclusive instruction through organization of these aspects into a sequence which can be duplicated and ensure clarity, uniformity and flexibility in education settings.

## Method details

The SENSE-L Framework is a systematic pedagogical system, which can be divided into five consecutive phases, which help learn the language by the auditory and sensory channels. The framework is oriented to make it replicable and can be scaled to alternative educational tiers and in classroom contexts.

Stage 1: Sensory Substitution of input.

Here, visual text is substituted with other sensory inputs. Content can be presented in the form of audio narration, oral reading or Braille. The pacing and repetition of the delivery are done so that they can understand and provide the learners with a chance to get language both visually and through feeling.

Stage 2: Linguistic Decomposition

The input is also carefully divided into smaller linguistic units such as phonemes, words and sentence structures. This breakdown ensures that the learners will have the ability to digest the language bit by bit and consolidate the knowledge by repetitions and listening comprehension.

Stage 3: Sensory Anchoring

Here, linguistic aspects relate to sensory inputs, which include the tone, rhythm and experiences. These associations enhance the recollection and help the learners to relate the language with sensory perception.

Stage 4: Guided Interpretation

Students are supported by using step-by-step prompts that help to make interpretations. Analytical skills are developed with the help of the questions about the meaning, tone and emotional response. Emphasis is put on verbal interaction to facilitate thinking.

Stage 5: Expressive Output

At the last step, students create a language by means of oral narration, audio recordings, or writing in Braille. It focuses on an organized articulation, which allows the learners to express the understanding in a logical and systematic way.

The qualitative validity of these stages was tested by qualitative interview-based validation, both between the learners and the instructors as presented in the next section.

## Method validation

Interview responses were transcribed, coded, and grouped according to the five stages of the SENSE-L Framework to enable systematic interpretation. The SENSE-L Framework validation involved an exploratory, mixed-method, mainly qualitative approach with eight visually impaired language learners (R1 through R8) and eight language teachers (T1 through T8) in an inclusive classroom. This validation focused on determining the working efficiency, accessibility and pedagogic suitability of the framework of five simple steps: sensory input substitution, linguistic decomposition, sensory anchoring, guided interpretation and expressive output.

The subjects were engaged in a structured learning session conducted based on the SENSE-L Framework. After delivery, a series of semi-structured interviews were held to elicit participant responses regarding the comprehension, retention, participation and confidence of the teaching session. Interview-based validation is consistent with current practice in research into inclusive pedagogy, in which experiential and perception-based data are imperative for assessing the impact of accessibility-focused strategies [1].

Alongside qualitative data, student responses were classified to produce suggestive quantitative response patterns. The combination of qualitative insight with proportional representations is in line with mixed-methods strategies used in educational research to account for greater interpretability while avoiding the reliance on inferential statistics [9].

Each participant's responses were classified as positive, mixed, or limited effect, and proportional analyses were computed for the total number of participants (n = 16). For the sensory input substitution stage, 12 participants (75%) out of 16 reported that auditory approach was effective in comprehension, while 4 participants (25%) felt that auditory only delivery of complex language features such as spelling and unfamiliar vocabulary were not enough to facilitate comprehension. This observation is consistent with the need to combine the auditory and tactile senses to get the best learning outcomes for visually impaired students [10].

During linguistic breaking down, all 16 participants (100%) reported better comprehension when language input was broken down into fragments like phonemes, words and clauses. This finding is consistent with research showing that linguistic decomposition decreases the cognitive load and increases efficiency [11]. In terms of sensory anchoring, 13 participants (81.25%) reported enhanced memory recall when repetition, rhythm and tone variations were used for instruction, with 3 participants (18.75%) reporting satisfactory learning gains. This echoes studies revealing the importance of auditory structure in memory processes [12].

During the interpretative prompting stage, all learners (8/8; 100%) and 7 teachers out of 8 (87.5%) revealed that the use of probing questions, and conferencing, promoted insight into meaning. This aligns with evidence of the role of scaffolds in facilitating interpretive activity [13]. In the expressive response stage, 14 participants (87.5%) reported improved confidence speaking and expressing responses and 2 participants (12.5%) reported a preference for indivualised expression, some preferring speaking and others Braille. This shows variability in learners' preferences, with the need for multiple response channels in education.

Qualitative data analysis adhered to a structured thematic analysis, in line with the five stages of the SENSE-L Framework. Transcriptions of the interview data were inductively coded to produce codes, including accessibility, understanding, retention, engagement, confidence, and preferred mode. They were then collated into themes suitable for each stage of the framework.

To increase the analysis' rigor, triangulation was used by examining differences and similarities between learners and teachers, noting convergent and divergent responses. The convergent themes related uniquely to a better understanding through language segmentation, increased retention through repetition and increased engagement through questioning. Divergent themes were related to expressive preferences, reflecting the uniqueness of learners. Learner testimonies can offer more light. For example, learners commonly referred to the benefits of segmentation in easing cognitive load, with one participant saying "the lesson was broken down into smaller chunks to follow step by step" (R4). Likewise, the importance of repetition to memory consolidation was also noted, with another participant noting that "hearing the same phrases again helped me remember them clearly" (R1). These findings were also supported by teacher feedback, with teachers noting that "students were more attentive and clarified their responses" (T2), and that "guided questions enhanced their questioning and meaning-making skills" (T3). Consequently, the thematic study shows that the framework not only maximizes access, but also leads to higher levels of cognition in literature and language.

The use of the proportional responses mixed with the thematic analysis is a strong confirmation for the SENSE-L Framework. The strong consensus evident among the learners, especially in the first two steps of linguistic decomposition and guided interpretation, suggests that the step-by-step processing of text is a key factor in improving comprehension and learning engagement among the visually impaired. The results also highlight the need for multi-sensory processing, with auditory information being the primary access point, although tactile feedback is beneficial. The diversity in preferred expression forms also emphasises the importance of flexible expression tools in inclusive education practices. In conclusion, the agreement among eight learners and eight teachers validate the reliability of the findings, and suggest that the SENSE-L Framework can be used as a replicable framework for inclusive language development and literature teaching.

Stage 1: Sensory Substitution of input

The input feedback from these learners suggests that auditory delivery of course content was successful, with all learners reporting they could understand the content. But several participants noted issues with the lack of tactile support. R1′s struggles with spelling, for example, and R3′s and R5′s preference to use Braille to understand structure illustrates this. It would appear that while delivery is supported auditory, multimodal reinforcement is required for full understanding of language.

Stage 2: Linguistic Decomposition

Virtually all learners agreed to better comprehend content decomposed into smaller segments. The descriptives of "less confusion" (R4) and "less clutter" (R5) suggest the deep processing made available through linguistic decomposition helps understanding.

Stage 3: Sensory Anchoring

Respondents indicate that repetition and rhythm are crucial to remembering. Participants reported better retention as a result of repetition, validating the use of sensory anchoring.

Stage 4: Guided Interpretation

Students universally found the interpretation promoted by targeted questioning assisted in better understanding, supporting the use of scaffolded interpretation.

Stage 5: Expressive Output

Students all reported a higher confidence in their abilities to express through Braille and speech compared to earlier lessons.

**Table:** 

**Framework Stage**	**Key Theme**	**Quantitative Indicator**	**Qualitative Evidence**
Sensory Input	Accessible with tactile limitation	75% positive; 25% partial	“Audio clear, but needs touch for spelling” (R1)
Decomposition	Strong comprehension improvement	100% positive	“Breaking into parts reduced confusion” (R4)
Anchoring	Enhanced retention	81.25% positive	“Repetition helped memory” (R1)
Interpretation	Improved meaning-making	100% learners; 87.5% teachers	“Questions guided understanding” (R2)
Expression	Increased confidence with variation	87.5% positive	“Students more confident in responses” (T3)

Teacher responses indicate that the SENSE-L Framework is pedagogically feasible and enhances classroom engagement. All instructors reported improved student participation, with T2 noting increased involvement among quieter learners. Comprehension gains were also observed, with teachers reporting clearer and more structured student responses. While the method was considered practical, several instructors noted the need for additional preparation time and suggested incorporating teaching aids and interactive elements for improved implementation.

## Limitations

The study is limited by its reliance on qualitative interview-based data bearing a small sample size with a small group of participants, which may limit the ability to apply the findings broadly to a wider population. Additionally, the absence of longitudinal measurement limits the assessment of sustained learning outcomes. Variability in access to tactile resources may also influence the implementation of the framework across different contexts.

## Ethics statements

This study involved human participants. Informed consent was obtained from all participants, and all data were anonymized prior to analysis.

## CRediT author statement

David Paul: Conceptualization, Methodology, Validation

Daisy Gohain: Validation & review

Thejas Gigy: Writing – Original Draft Preparation

Binu Jerlin Harini: Writing – Review & Editing.

Supplementary material and/or additional information [OPTIONAL]

## Related research article

None.

## Declaration of competing interest

The authors declare that they have no known competing financial interests or personal relationships that could have appeared to influence the work reported in this paper.

## Data Availability

No data was used for the research described in the article.
